# Supplementary material for: Integrating evolutionary dynamics into treatment of metastatic castrate-resistant prostate cancer
Source: Nat Commun. 2017 Nov 28;8:1816. doi: 10.1038/s41467-017-01968-5 (PMC5703947; doi:10.1038/s41467-017-01968-5)
Supplement: Supplementary file 1 — Supplementary Information [file 41467_2017_1968_MOESM1_ESM.pdf]

	ESS's k_i=25000			ESS's k_i = [1.5*TP, 10000, 10000]		
	**T+**	**TP**	**T-**	**T+**	**TP**	**T-**
**9**	55%	45%	0%	50%	50%	0%
**10**	55%	45%	0%	50%	50%	0%
**15**	55%	45%	0%	50%	50%	0%
**16**	45%	55%	0%	50%	50%	0%
**22**	45%	55%	0%	44%	56%	0%
**13**	45%	55%	0%	47%	53%	0%
**14**	40%	60%	0%	47%	53%	0%
**21**	40%	60%	0%	47%	53%	0%
**7**	33%	66%	0%	52%	48%	0%
**8**	55%	45%	0%	52%	48%	0%
**20**	45%	55%	0%	52%	48%	0%
**19**	40%	60%	0%	52%	48%	0%
**12**	37%	57%	7%	48%	50%	2%
**5**	41%	52%	7%	45%	52%	3%
**2**	29%	63%	9%	41%	55%	3%
**6**	45%	42%	13%	38%	55%	7%
**18**	20%	63%	17%	33%	47%	20%
**1**	25%	50%	25%	25%	54%	21%
**4**	17%	58%	25%	22%	51%	27%
**3**	28%	40%	32%	0%	40%	60%
**11**	33%	33%	33%	0%	40%	60%
**17**	17%	37%	47%	0%	40%	60%

Supplementary Table 1: Sensitivity analysis of carrying capacities. $k = [1.5 * TP, \phi, \phi]$ where ϕ can be any value results in the same ESS's. A more detailed analysis of simulation sensitivity analysis in Li et al.

	$\boldsymbol{\alpha} = [\mathbf{0.1\ 0.2\ 0.3\ 0.4\ 0.5\ 0.6}]$			$\boldsymbol{\alpha} = [\mathbf{0}.0\ 0.2\ 0.4\ 0.6\ 0.8\ 1.0]$		
	**T+**	**TP**	**T-**	**T+**	**TP**	**T-**
**9**	52%	45%	3%	52%	48%	0%
**10**	55%	45%	0%	52%	48%	0%
**15**	56%	43%	1%	57%	43%	0%
**16**	57%	43%	0%	57%	43%	0%
**22**	58%	42%	0%	60%	40%	0%
**13**	48%	41%	11%	57%	43%	0%
**14**	49%	42%	9%	57%	43%	0%
**21**	52%	40%	8%	60%	40%	0%
**7**	44%	45%	12%	47%	53%	0%
**8**	46%	44%	10%	52%	48%	0%
**20**	46%	39%	15%	60%	40%	0%
**19**	44%	38%	18%	43%	29%	29%
**12**	40%	40%	20%	57%	43%	0%
**5**	39%	41%	21%	52%	48%	0%
**2**	38%	43%	19%	47%	53%	0%
**6**	38%	44%	18%	47%	53%	0%
**18**	38%	37%	25%	60%	40%	0%
**1**	31%	43%	26%	44%	53%	3%
**4**	31%	40%	28%	51%	48%	1%
**3**	21%	38%	41%	0%	33%	67%
**11**	29%	38%	34%	0%	33%	67%
**17**	27%	35%	37%	0%	33%	67%

Supplementary Table 2: Effect of varying values of $\boldsymbol{\alpha_{ij}}$ on variance and coexistence of ESS's.

	Time to Progression 5% T-			Time To Progression 90% T-		
	**MTD**	**Metronomic**	**Adaptive**	**MTD**	**Metronomic**	**Adaptive**
**9**	---	---	---	---	---	---
**10**	---	---	---	---	---	---
**15**	---	---	---	---	---	---
**16**	---	---	---	---	---	---
**22**	---	---	---	---	---	---
**13**	6361	6898	---	7005	7765	---
**14**	6254	6828	---	6908	7750	---
**21**	7069	7760	---	7718	8717	---
**7**	3950	4556	8557	4621	4950	---
**8**	4378	4832	9141	5047	5757	---
**20**	902	902	2046	1456	1456	3132
**19**	759	759	915	1036	1036	2029
**12**	673	673	673	802	802	4126
**5**	323	323	323	784	784	---
**2**	161	161	161	759	759	1888
**6**	1	1	1	679	679	1596
**18**	1	1	1	560	560	560
**1**	1	1	1	532	532	532
**4**	1	1	1	501	501	501
**3**	1	1	1	334	334	334
**11**	1	1	1	334	334	334
**17**	1	1	1	334	334	334

Supplementary Table 3: Time to progression analysis for the three treatment regimens on all 22 possible tumor initial conditions. Representative patient #1 corresponds to **7** and representative patients #2 corresponds to **5** (highlighted). It can be seen that that non-responders category at the bottom shows no difference in time to progression for any treatments. In all other initial conditions the adaptive therapy regimen provides equivalent or longer times to progression.
